# Unlocking microglia pyroptosis in a model of type I interferon-driven neuroinflammation: lessons from Rnaset2^−/−^ mice

**DOI:** 10.1038/s41419-025-08350-0

**Published:** 2025-12-27

**Authors:** Kristin Wendland, Milena Irsfeld, Kathrin Schreiber, Katharina Ternka, Christine Stadelmann, Stefan Nessler, Jutta Gärtner, Matthias Kettwig

**Affiliations:** 1 Department of Pediatrics and Adolescent Medicine, Division of Pediatric Neurology, University Medical Center Göttingen, Göttingen, Germany; 2 German Center for Child and Adolescent Health (DZKJ), Göttingen, Germany; 3 Department of Neuropathology, University Medical Center Göttingen, Göttingen, Germany

**Keywords:** Paediatric neurological disorders, Neuroimmunology, Cell death, Cell death and immune response, Translational research

## Abstract

RNaseT2-deficient cystic leukoencephalopathy (CLE) presents with severe psychomotor retardation, cystic brain lesions, white matter alterations, and cerebral atrophy. The *Rnaset2*^*−/−*^ mouse mirrors key features of this disease and represents the first murine model with a distinct neurological phenotype for type I interferonopathies. *Rnaset2*^*−/−*^ mice exhibit activated microglia, perivascular monocyte and CD8 + T cell infiltration, and hippocampal accentuated atrophy. However, the mechanisms linking interferon-driven neuroinflammation to neurodegeneration remain unclear, underscoring the need to clarify which molecular processes contribute to tissue injury in a time-dependent manner. We found a sustained upregulation of interferon-stimulated genes (IRF9, RIG-I) over three to 28 weeks of age in the brains of *Rnaset2*^*−/−*^ mice compared to controls. Expression of the chemokines *Ccl2, Ccl5*, and *Cxcl10* peaked early but declined thereafter. Pyroptosis-related markers (ASC, CASP1, GSDMD) were significantly increased already at three to 6 weeks of age and decreased thereafter, whereas apoptotic markers such as *Bax, Bad, Bid,* CASP3, CASP8, and PARP were not differentially expressed compared to controls. Finally, *Cd3e* as well as *Tnf* peaked later (at 17 weeks of age) and declined at 28 weeks. Interestingly, double IHC confirmed the co-localization of the pyroptosis-related marker ASC with the microglia marker IBA-1. Taken together, these findings support the notion that pyroptosis is an early, disease-associated event restricted to microglia that likely contributes to establishing a proinflammatory milieu prior to T cell infiltration and brain atrophy. Targeting pyroptosis could therefore represent a potential strategy to attenuate neurodegeneration in type I interferon–driven neuroinflammatory disorders.

## Introduction

RNaseT2-deficient cystic leukoencephalopathy (CLE) is a rare, autosomal recessive disorder caused by loss-of-function mutations in the *RNASET2* gene. It manifests in early childhood with psychomotor delay, spasticity, epilepsy, and normo- or microcephaly. Brain magnetic resonance imaging (MRI) reveals characteristic cystic lesions in the temporal and frontal lobes, multifocal abnormalities of white matter, and cerebral atrophy [1, 2]. The clinical and radiological features of RNaseT2-deficient CLE closely resemble those observed in congenital cytomegalovirus (CMV) infection and Aicardi–Goutières syndrome (AGS), a group of genetically heterogeneous type I interferonopathies characterized by early-onset progressive encephalopathy [3, 4]. AGS presents with microcephaly, severe developmental delay, spasticity, and seizures; neuroimaging typically reveals intracranial calcifications, white matter abnormalities, and cerebral atrophy. Systemic signs may include chilblain-like skin lesions and chronic inflammation [2, 5]. These similarities suggest shared pathophysiological mechanisms driven by dysregulated type I interferon (IFN-I) signaling. IFN-I functions as a key antiviral cytokine, inducing antiviral effector genes, inhibiting cellular processes, and promoting T cell expansion [6, 7]. However, chronic IFN-I stimulation can lead to sustained neuroinflammation and tissue damage [8, 9].

Mouse models of interferonopathies often incompletely recapitulate human neuroinflammation [10]. To investigate shared mechanisms of RNaseT2-deficient CLE, congenital CMV infection, and AGS, we recently established the first mouse model of RNaseT2-deficient CLE, which develops IFN-I–driven neuroinflammation characterized by microglial activation, leukocyte infiltration, blood–brain barrier dysfunction, and hippocampal accentuated atrophy [11]. While this model revealed that neuroinflammation and brain atrophy depend on IFN-I signaling, the molecular pathways linking sustained interferon activation to tissue pathology remain unclear.

To characterize the progression of IFN-I–driven neuroinflammation and tissue dysfunction in *Rnaset2*^*−/−*^ mice, we initially analyzed the temporal kinetics of interferon-stimulated gene (ISG) expression in brain tissue compared to *Rnaset2*^*+/+*^ controls at 3, 6, 17, and 28 weeks of age. Next, to elucidate the downstream mechanisms potentially responsible for the reported brain-atrophy phenotype in *Rnaset2*^*−/−*^ mice, we further investigated cell-death pathways, focusing on apoptosis and pyroptosis. Apoptosis was selected due to its well-characterized role as a form of programmed cell death, frequently associated with both developmental and pathological processes, including neurodegenerative disorders [12]. Pyroptosis, by contrast, is an inflammation-driven form of cell death marked by inflammasome activation through the adapter protein apoptosis-associated speck-like protein containing a caspase recruitment domain (ASC, mRNA: *Pycard*), which recruits and activates caspase-1 (CASP1) via its caspase recruitment domain (CARD) [13, 14]. Activated CASP1 cleaves gasdermin D (GSDMD), whose N-terminal fragment forms membrane pores, leading to the release of pro-inflammatory cytokines, including interleukin-1β (IL-1β) [15]. Furthermore, inflammatory mediators were quantified by qPCR to map the onset and progression of neuroinflammation, including *Tnf* (TNF-α), a key pro-inflammatory cytokine that amplifies immune activation and orchestrates inflammatory processes [16, 17], and the chemokines *Ccl2*, *Ccl5*, and *Cxcl10*, which drive leukocyte recruitment into the CNS [18–22]. Finally, mRNA expression of the T cell marker *Cd3e* was measured to evaluate immune cell infiltration.

This study aimed to investigate the temporal relationship between the induction of ISGs, the release of cytokines and chemokines, and the infiltration of immune cells (particularly T cells), as well as apoptosis- and pyroptosis-related mechanisms, to increase our knowledge of the pathophysiological processes underlying interferon-mediated neuroinflammation and neurodegeneration in RNaseT2-deficient CLE.

## Results

### Sustained upregulation of interferon-stimulated genes *Irf9* and *Ddx58* in *Rnaset2*^*−/−*^ mouse brains

**Fig. 1:**
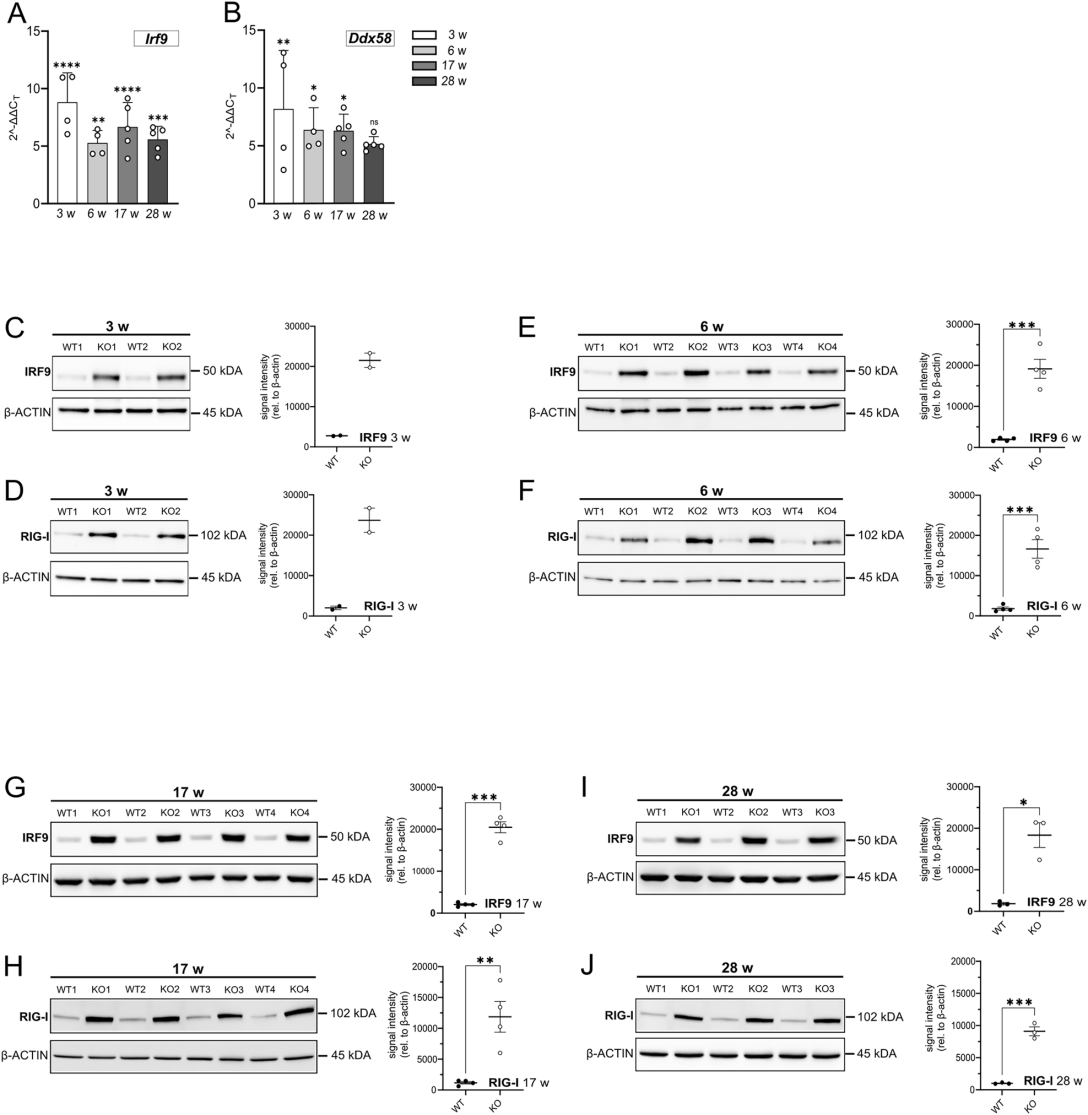
Sustained upregulation of interferon-stimulated genes *Irf9* and *Ddx58* in *Rnaset2*^−/−^ mouse brains. **A** mRNA expression levels of *Irf9* were quantified by qPCR in brain samples from *Rnaset2*^*−/−*^ and *Rnaset2*^*+/+*^ mice at 3, 6, 17, and 28 weeks of age. Values remain persistently increased, with mean fold changes between five and ten, showing no temporal peaks. Mean ± SEM with ^**^ = p < 0.01, ^***^ = p < 0.001, ^****^ = p < 0.0001, One-way ANOVA followed by Tukey’s multiple comparison test; *n* = *4* for 3 and 6 weeks, *n* = *5* for 17 and 28 weeks. *Irf9: interferon regulatory factor 9*. **B** mRNA expression levels of *Ddx58* were quantified by qPCR in brain samples from *Rnaset2*^*−/−*^ and *Rnaset2*^*+/+*^ mice at 3, 6, 17, and 28 weeks of age. Values consistently show elevated levels, with mean fold changes ranging from five to ten, demonstrating no temporal peaks. Mean ± SEM with ^*^ = p < 0.05, ^**^ = p < 0.01, ns not significant, One-way ANOVA followed by Tukey’s multiple comparison test; *n* = *4* for 3 and 6 weeks, *n* = *5* for 17 and 28 weeks. *Ddx58: DEAD-Box Helicase 58*. **C** Western blot analysis of IRF9 in *Rnaset2*^*−/−*^ brains compared to *Rnaset2*^*+/+*^ at 3 weeks of age. Blot intensity differences clearly depict increased IRF9 protein amounts. Likewise, densitometry quantification shows a substantial grey value difference of over 15,000 grey values between *Rnaset2*^*−/−*^ and *Rnaset2*^*+/+*^ samples. Loading control: β-ACTIN. Mean ± SEM; *n* = *2* per group and time point. WT1-2: *Rnaset2*^*+/+*^, KO1-2: *Rnaset2*^*−/−*^. IRF9: interferon regulatory factor 9. **D** Western blot analysis of RIG-I in *Rnaset2*^*−/−*^ brains compared to *Rnaset2*^*−/−*^ at 3 weeks of age. Differences in RIG-I grey value number are clearly visible between *Rnaset2*^*−/−*^ and *Rnaset2*^*+/+*^ protein samples. Likewise, densitometry quantification demonstrates a 15,000-fold difference in grey values between *Rnaset2*^*−/−*^ and *Rnaset2*^*+/+*^ protein samples. Loading control: β-ACTIN. Mean ± SEM; *n* = *2* per group and time point. WT1-2: *Rnaset2*^*+/+*^, KO1-2: *Rnaset2*^*−/−*^. RIG-I: Retinoic acid-inducible gene I. **E** Western blot analysis of IRF9 protein amount at 6 weeks of age. Densitometry analysis of IRF9 signal intensities reveals a significant increase in IRF9 protein amounts in *Rnaset2*^*−/−*^ brain protein samples compared to samples from *Rnaset2*^*+/+*^ (10-fold increase in *Rnaset2*^*−/−*^, p < 0.001). Loading control: β-ACTIN. Mean ± SEM with ^***^ = p < 0.001, Student’s *t* test; *n* = *4* per group and time point. WT1-4: *Rnaset2*^*+/+*^, KO1-4: *Rnaset2*^*−/−*^. IRF9: interferon regulatory factor 9. **F** Western blot analysis of RIG-I at 6 weeks of age. Densitometry analysis of RIG-I signal intensities depicts a significant upregulation in RIG-I protein amounts in *Rnaset2*^*−/−*^ brains compared to *Rnaset2*^*+/+*^ (nine-fold increase in *Rnaset2*^*−/−*^, p < 0.001). Loading control: β-ACTIN. Mean ± SEM with ^***^ = p < 0.001, Student’s *t*-test; *n* = *4* per group and time point. WT1-4: *Rnaset2*^*+/+*^, KO1-4: *Rnaset2*^*−/−*^. RIG-I: Retinoic acid-inducible gene I. **G** Western blot analysis of IRF9 at 17 weeks of age. Densitometry quantification of IRF9 signal intensities reveals a significant increase in IRF9 in *Rnaset2*^*−/−*^ brains compared to *Rnaset2*^*+/+*^ (ten-fold increase in *Rnaset2*^*−/−*^, p < 0.001). Loading control: β-ACTIN. Mean ± SEM with ^***^ = p < 0.001, Student’s *t* test; *n* = *4* per group and time point. WT1-4: *Rnaset2*^*+/+*^, KO1-4: *Rnaset2*^*−/−*^. IRF9: interferon regulatory factor 9. **H** Western blot analysis of RIG-I at 17 weeks of age. In comparison to *Rnaset2*^*+/+*^ brain samples, densitometry analysis of RIG-I signal intensities shows a significant increase in RIG-I protein amount in *Rnaset2*^*−/−*^ protein samples (10-fold increase in *Rnaset2*^*−/−*^, p < 0.01). Loading control: β-ACTIN. Mean ± SEM with ^**^ = p < 0.01, Student’s *t* test; *n* = *4* per group and time point. WT1-4: *Rnaset2*^*+/+*^, KO1-4: *Rnaset2*^*−/−*^. RIG-I: Retinoic acid-inducible gene I. **I** Western blot analysis of IRF9 at 28 weeks of age. Blot intensities were measured using densitometry, and results show significantly higher levels of IRF9 protein in *Rnaset2*^*−/−*^ samples (ten-fold increase in *Rnaset2*^*−/−*^, p < 0.05). Loading control: β-ACTIN. Mean ± SEM with ^*^ = p < 0.05, Student’s *t* test; *n* = *3* per group and time point. WT1-3: *Rnaset2*^*+/+*^, KO1-3: *Rnaset2*^*−/−*^. IRF9: interferon regulatory factor 9. **J** Western blot analysis of RIG-I at 28 weeks of age. Blot densitometry shows significantly upregulated RIG-I protein amounts in *Rnaset2*^*−/−*^ brain samples (nine-fold increase in *Rnaset2*^*−/−*^, p < 0.001). Loading control: β-ACTIN. Mean ± SEM with ^***^ = p < 0.001, Student’s *t* test; *n* = *3* per group and time point. WT1-3: *Rnaset2*^*+/+*^, KO1-3: *Rnaset2*^*−/−*^. RIG-I: Retinoic acid-inducible gene I.

We recently showed an increase in ISG expression in the brains of *Rnaset2*^*−/−*^ mice [11]. However, the precise onset of ISG upregulation has yet to be identified. Since persistent type I IFN signaling is thought to drive subsequent inflammatory processes, it was important to first define the temporal dynamics of ISG expression in detail. This allowed us to determine whether the onset and persistence of ISG induction correlate with later stages of neuroinflammation. Employing qPCR, we analyzed the mRNA levels of interferon regulatory factor 9 (*Irf9*) and DEAD box protein 58 (*Ddx58*) in both *Rnaset2*^*−/−*^ and *Rnaset2*^*+/+*^ mice brains at 3, 6, 17, and 28 weeks after birth. We found that *Irf9* and *Ddx58* mRNA levels were significantly increased at 3 weeks in *Rnaset2*^*−/−*^ brains (*Irf9:* fold change (FC) 8.8 ± 1.3; *Ddx58:* FC 8.1 ± 2.5) compared to *Rnaset2*^*+/+*^ mice, slightly decreased at 6 weeks (*Irf9:* FC 5.3 ± 0.5; *Ddx58:* FC 6.3 ± 1.0), and maintained elevated thereafter (Fig. 1A, B). These results indicate persistent interferon signaling in *Rnaset2*^*−/−*^ brains. To further substantiate the interferon signature and to enable comparison with other disease models such as Aicardi–Goutières syndrome, we also assessed the established ISG panel commonly used to calculate the interferon score in blood *(Ifi27l2a, Ifi44, Ifit1, Isg15, Rsad2*, and *Siglec1)*. This analysis revealed a consistent and robust ISG upregulation in *Rnaset2*^*−/−*^ brains across the measured time points (Supplementary Fig. A and B). However, these transcripts showed increased variability among samples and time points. Consequently, this investigation further concentrated on *Irf9* and *Ddx58* for additional examinations.

IRF9 and RIG-I (*Ddx58* mRNA) protein levels were increased in all *Rnaset2*^*−/*−^ animals at 3, 6, 17, and 28 weeks, as illustrated in Fig. 1C–J. Densitometry analysis revealed significant differences in IRF9 and RIG-I protein levels between *Rnaset2*^*−/−*^ *and Rnaset2*^*+/+*^ mice at 6 weeks (ten-fold increase for IRF9 and 9-fold increase for RIG-I), 17 weeks (ten-fold increase for both IRF9 and RIG-I), and 28 weeks of age (10-fold increase for IRF9 and nine-fold increase for RIG-I) (Fig. 1E–J). These results align with the transcriptional data, likewise indicating sustained activation of the interferon pathway in the absence of RNASET2.

### Initial and transient upregulation of apoptotic marker mRNA in *Rnaset2*^*−/−*^ mouse brains was not corroborated at the protein level

One of the key features of interferon-driven neuroinflammation is brain atrophy, especially in the hippocampus. Since apoptosis and pyroptosis are the two most plausible cell death routes in this condition, we set out to investigate whether they contribute to the neurodegenerative phenotype of RNaseT2-deficient CLE.

**Fig. 2:**
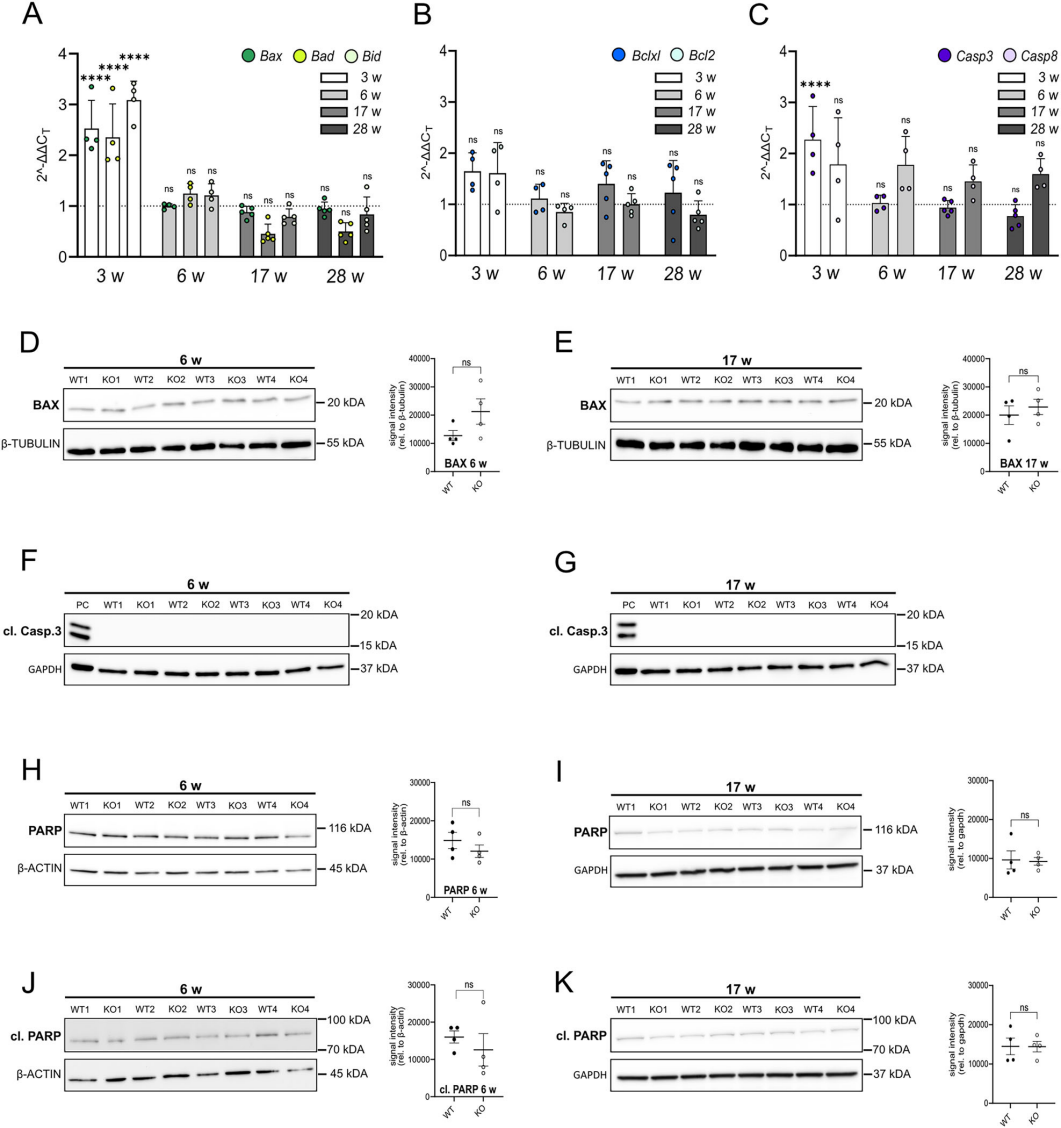
Initial and transient upregulation of apoptotic marker mRNA in *Rnaset2*^*−/−*^ mouse brains was not corroborated at the protein level. **A** The 2^−ΔΔCt^ method was employed to analyze qPCR experiments of pro-apoptotic markers *Bax*, *Bad*, and *Bid* in brain samples at 3, 6, 17, and 28 weeks of age. Significant upregulation of *Bax*, *Bad*, and *Bid* was observed in *Rnaset2*^*−/−*^ mice at 3 weeks in comparison to *Rnaset2*^*+/+*^ mice, with a mean fold change slightly exceeding 2.0 (p < 0.0001). However, no significant differences between *Rnaset2*^*−/−*^ and *Rnaset2*^*+/+*^ mice were observed at subsequent time points (6, 17, and 28 weeks). Data are presented as scatter dot plots with mean ± SEM with ^****^ = p < 0.0001, ns not significant, One-way ANOVA followed by Tukey’s multiple comparison test; Analysis of *Bax* expression: Kruskal–Wallis test with Dunn’s multiple comparisons test; *n* = *4* for 3 and 6 weeks; *n* = *5* for 17 and 28 weeks. The dotted line depicts a fold change of one. *Bax Bcl-2-associated X protein, Bad Bcl-2-associated death promoter, Bid BH3-interacting domain death agonist*. **B** Expression analysis of anti-apoptotic markers *Bclxl* and *Bcl2* at 3, 6, 17, and 28 weeks. Scatter dot plots indicate no significant upregulation at any age. Data are presented as scatter dot plots with mean ± SEM with ns not significant, One-way ANOVA followed by Tukey’s multiple comparison test; *n* = *4* for 3 and 6 weeks; *n* = 5 for 17 and 28 weeks. Dotted line depicts a fold change of one. *Bclxl B-cell lymphoma-extra large*, *Bcl2* *B-cell lymphoma 2*. **C** qPCR analysis of mRNA expression of *Casp3* and *Casp8* in *Rnaset2*^*−/−*^ and *Rnaset2*^*+/+*^ brain samples. Significant upregulation of *Casp3* is observed in *Rnaset2*^*−/−*^ mice at 3 weeks, with a mean fold change just above 2.0 (p < 0.0001). At 6, 17, and 28 weeks, no significant differences were detected for either *Casp3* or *Casp8* between *Rnaset2*^*−/−*^ and *Rnaset2*^*+/+*^ mice brains. Scatter dot plots with mean ± SEM with ^****^ = p < 0.0001, ns not significant, One-way ANOVA followed by Tukey’s multiple comparison test; *n* = *4* for 3 and 6 weeks; *n* = *5* for 17 and 28 weeks for *Casp3*, and *n* = *4* for 17 and 28 weeks for *Casp8*. Dotted line depicts a fold change of one. *Casp3 caspase 3, Casp8 caspase8*. Western blot analysis for BAX protein in brain tissues at 6 (**D**) (*n* = *4* per genotype) and 17 weeks (**E**) (*n* = *4* per genotype, (**E**)). No significant differences in BAX protein expression were detected at either time point. Loading control: β-TUBULIN; Mean ± SEM with ns not significant, Student’s *t* test; *n* = *4* per group and time point. WT1-4: *Rnaset2*^*+/+*^, KO1-4: *Rnaset2*^*−/−*^. BAX: Bcl-2-associated X protein. Western blot analysis of cleaved CASPASE 3 (cl. CASP3) in brain tissue at 6 (**F**) and 17 weeks (**G**). Cleaved CASP3 was undetectable in both *Rnaset2*^*−/−*^ and *Rnaset2*^*+/+*^ samples, suggesting the absence of ongoing apoptosis in a relevant proportion of cells. As a positive control, Caspase 3 control cell extracts (#9663, Cell Signaling Technology) were included and yielded the expected band. GAPDH served as loading control. Data are shown as mean ± SEM; *n* = 4 per group and time point. WT1–4: *Rnaset2*^*+/+*^, KO1–4: *Rnaset2*^*−/−*^. cl. CASP3 cleaved Caspase 3. Western blot analysis for full-length PARP at 6 (**H**) and 17 weeks (**I**) revealed no significant difference in protein amounts between *Rnaset2*^*−/−*^ and *Rnaset2*^*+/+*^ brain samples. This result indicates the absence of apoptosis in a substantial number of cells. Loading control: β-ACTIN and GAPDH. Data are shown as mean ± SEM with ns not significant, Student’s *t* test; *n* = *4* per group and time point. WT1-4: *Rnaset2*^*+/+*^, KO1-4: *Rnaset2*^*−/−*^. PARP: Poly (ADP-Ribose) Polymerase 1. At 6 (**J**) and 17 weeks (**K**), Western blot analysis revealed that cleaved PARP (cl. PARP) exhibits no discernible difference in protein amounts between *Rnaset2*^*−/−*^ and *Rnaset2*^*+/+*^ brain samples. Loading control: β-ACTIN and GAPDH. Data are shown as mean ± SEM with ns not significant, Student’s *t* test; *n* = *4* per group and time point. WT1-4: *Rnaset2*^*+/+*^, KO1-4: *Rnaset2*^*−/−*^. cl. PARP cleaved Poly (ADP-Ribose) Polymerase 1.

Figure 2A presents the mRNA expression levels of the pro-apoptotic markers *Bax*, *Bad*, and *Bid* at 3, 6, 17, and 28 weeks. We found a transient increase of pro-apoptotic transcripts at 3 weeks in *Rnaset2*^*−/−*^ brains (*Bax:* FC 2.5 ± 0.3; *Bad:* FC 2.4 ± 0.3; *Bid:* FC 3.1 ± 0.2; all p < 0.0001), with no significant increase observed at 6, 17, or 28 weeks (Fig. 2A). Anti-apoptotic transcripts (*Bclxl, Bcl2*) were not elevated at any time point, indicating no compensatory anti-apoptotic response (Fig. 2B).

Caspase 3 (CASP3) and caspase 8 (CASP8) are key enzymes in apoptosis, with CASP3 serving as a primary executioner caspase [23] and CASP8 initiating death receptor-mediated apoptosis [24]. Given their roles, CASP3 and CASP8 are valuable markers to assess apoptotic activity in the brains of *Rnaset2*^*−/−*^ mice. Across all investigated time points, *Casp3* mRNA expression was significantly increased in *Rnaset2*^*−/−*^ compared to *Rnaset2*^*+/+*^ mice only at 3 weeks of age, with a fold change of 2.3 ± 0.3. At all other time points, *Casp3* mRNA levels showed no notable differences between *Rnaset2*^*−/−*^ and *Rnaset2*^*+/+*^ mice (Fig. 2C). Similarly, *Casp8* expression analysis revealed no significant differences between *Rnaset2*^*−/−*^ *and Rnaset2*^*+/+*^ mice throughout the measurement period (Fig. 2C). Consequently, these findings do not indicate ongoing apoptosis.

The qPCR data showed an increase in *Bax* and *Casp3* mRNA only at 3 weeks of age. To validate this result at the protein level, we quantified BAX and cleaved CASP3 protein expression in brains of *Rnaset2*^*−/−*^ and *Rnaset2*^*+/+*^ mice (Supplementary Fig. C and D). Although BAX levels were generally comparable, one *Rnaset2*^*−/−*^ animal showed a slight increase relative to *Rnaset2*^*+/+*^ controls at 3 weeks (Supplementary Fig. C). At 6 weeks, two *Rnaset2*^*−/−*^ animals exhibited elevated but not significant BAX protein levels (Fig. 2D), while at 17 weeks, no differences between genotypes were observed (Fig. 2E). Cleaved CASP3 was not detectable in any brain samples at 3 (Supplementary Fig. D), 6 (Fig. 2F), and 17 weeks (Fig. 2G). Finally, to further verify the absence of apoptosis, we examined changes in the protein levels of poly(ADP-ribose) polymerase 1 (PARP), a recognized apoptosis marker integral to DNA repair and cleaved during apoptosis (cleaved PARP) [25]. No differences in full-length PARP or cleaved PARP expression were detected at any of the investigated time points (Supplementary Fig. E, F and 2H–K). According to these results, apoptosis appears not to be induced in the brains of *Rnaset2*^*−/−*^ mice.

### Continuous upregulation of pyroptosis at both transcriptional and translational levels in *Rnaset2*^*−*^^*/−*^ mice brains

**Fig. 3:**
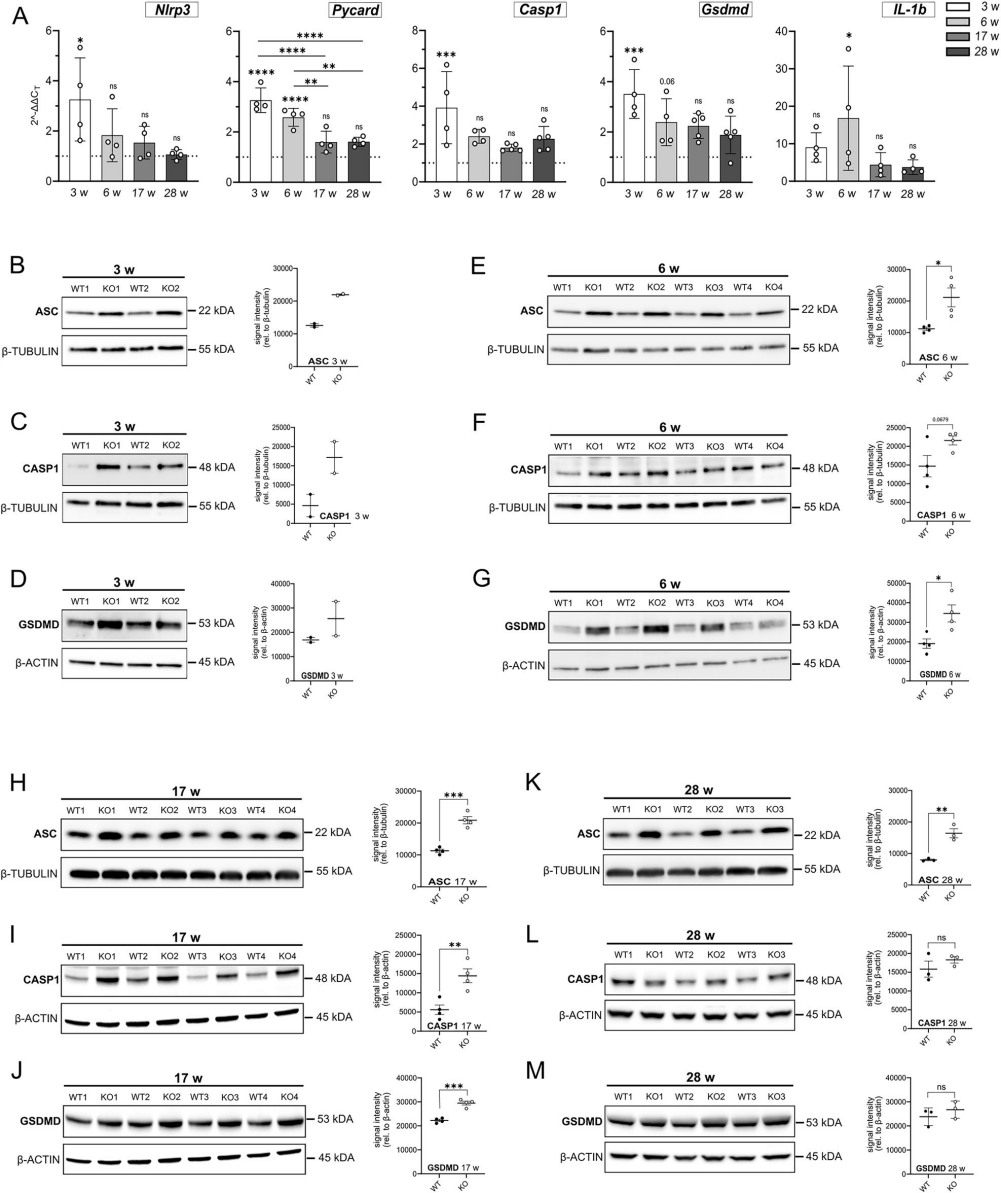
Continuous upregulation of pyroptosis at both transcriptional and translational levels in *Rnaset2*^−^^*/−*^ mice. **A** qPCR results for pyroptosis-related markers (*Nlrp3, Pycard (protein: ASC), Casp1, Gsdmd*, and *IL-1b*) in *Rnaset2*^*−/−*^ mouse brain samples, normalized to *Rnaset2*^*+/+*^ brain samples and a housekeeping gene at 3, 6, 17, and 28 weeks of age. *Nlrp3* mRNA expression exhibits a substantial increase at 3 weeks (p < 0.05), after which *Nlrp3* mRNA levels decline and remain low. The mean expression of *Asc (Pycard)* mRNA shows a significant increase at 3 weeks (p < 0.0001) and sustained upregulation at 6 weeks (p < 0.0001). Furthermore, *Pycard* mRNA levels at 3 and 6 weeks are significantly elevated (p < 0.0001) compared to 17 and 28 weeks, indicating an early pyroptotic response. *Casp1* and *Gsdmd* exhibit a comparable pattern, with both mRNAs significantly increased at 3 weeks (p < 0.001), aligning with a peak in pyroptotic activity. Similar to *Nlrp3*, both markers exhibit diminished expression levels at subsequent time points. *IL-1b*, a downstream effector of pyroptosis, shows a delayed response, with a significant increase occurring only at 6 weeks (p < 0.05) before declining. This pattern is distinct from the immediate early response of *Nlrp3*, *Pycard*, *Casp1*, and *Gsdmd*, emphasizing a subsequent cytokine response. Data are depicted as scatter dot plots with bars representing mean ± SEM with ^*^ = p < 0.05, ^**^ = p < 0.01, ^***^ = p < 0.001, ^****^ = p < 0.0001, ns not significant, One-way ANOVA followed by Tukey’s multiple comparison test; *n* = *4* for *Nlrp3, Asc (Pycard), IL-1b* for all measured time points; *n* = *4* for *Casp1* and *Gsdmd* at 3 and 6 weeks of age; *n* = *5* for *Casp1* and *Gsdmd* for 17 and 28 weeks of age. *Nlrp3: NOD-like receptor pyrin domain-containing 3, Asc: Apoptosis-associated speck-like protein containing a CARD, Casp1: Caspase 1, Gsdmd: Gasdermin D, IL-1β: Interleukin-1 beta*. (B-D) Western blot analysis of brain tissues from *Rnaset2*^*−/−*^ and *Rnaset2*^*+/+*^ mice demonstrates elevated ASC protein levels at 3 weeks of age, indicating inflammasome activation (**B**). Similarly, CASP1 protein levels are increased in *Rnaset2*^*−/−*^ brain specimens (**C**). GSDMD protein levels exhibit variation, evidenced by an increase in one of the two *Rnaset2*^*−/−*^ brain samples (**D**). Loading control: β-ACTIN or β-TUBULIN. Mean ± SEM; *n* = *2* per group and time point. WT1-2: *Rnaset2*^*+/+*^, KO1-2: *Rnaset2*^*−/−*^. ASC: Apoptosis-associated speck-like protein containing a CARD, CASP1: Caspase 1, GSDMD: Gasdermin D. (**E**–**G**) The 6-week time point reveals increased levels of pyroptotic markers in *Rnaset2*^−^^*/−*^ brain samples. ASC protein levels are persistently increased in *Rnaset2*^*−/−*^ samples, indicating continuous inflammasome activation (E, p < 0.05). CASP1 protein amounts show a trend toward increased levels in *Rnaset2*^*−/−*^ brain tissues (**F**). GSDMD protein levels are significantly increased (**G**, p < 0.05). Loading control: β-ACTIN or β-TUBULIN. Mean ± SEM with ^*^ = p < 0.05, CASP1: p = 0.0679 ns not significant, Student’s *t* test; *n* = *4* per group and time point. WT1-4: *Rnaset2*^*+/+*^, KO1-4: *Rnaset2*^*−/−*^. ASC: Apoptosis-associated speck-like protein containing a CARD, CASP1 Caspase 1, GSDMD Gasdermin D. **H**–**J** At 17 weeks, *Rnaset2*^*−/−*^ specimens display sustained activation of pyroptotic indicators. ASC protein levels are significantly elevated in *Rnaset2*^*−/−*^ brain samples (**H**, p < 0.001). Likewise, the protein amount of CASP1 is significantly increased in *Rnaset2*^*−/−*^ brain samples, signifying persistent pyroptotic signaling (**I**, p < 0.01). Similarly, GSDMD protein levels are significantly increased (**J**, p < 0.001). Loading control: β-ACTIN or β-TUBULIN. Mean ± SEM with ^**^ = p < 0.01, ^***^ = p < 0.001, Student’s *t* test; *n* = *4* per group and time point. WT1-4: *Rnaset2*^*+/+*^, KO1-4: *Rnaset2*^*−/−*^. ASC: Apoptosis-associated speck-like protein containing a CARD, CASP1 Caspase 1, GSDMD Gasdermin D. **K**–**M** The 28-week time point indicates a discontinuation of pyroptosis. The quantity of ASC protein remains elevated in *Rnaset2*^*−/−*^ brain samples (**K**, p < 0.01). CASP1 protein amounts exhibit no significant difference between *Rnaset2*^*−/−*^ and *Rnaset2*^*+/+*^ (**L**). Likewise, the quantity of GSDMD protein amounts shows no variation between *Rnaset2*^*−/−*^ and *Rnaset2*^*+/+*^ brain samples. Loading control: β-ACTIN or β-TUBULIN. Mean ± SEM with ^**^ = p < 0.01, ns not significant, Student’s *t* test; *n* = *3* per group and time point. WT1-3: *Rnaset2*^*+/+*^, KO1-3: *Rnaset2*^*−/−*^. ASC Apoptosis-associated speck-like protein containing a CARD, CASP1 Caspase 1, GSDMD Gasdermin D.

Since chronic neuroinflammation is a hallmark of *Rnaset2*^*−/−*^ mouse brains, we next focused on pyroptosis, a well-established inflammasome-dependent form of inflammatory cell death. We therefore conducted a comprehensive analysis of key pyroptotic markers at both the mRNA (Fig. 3A) and protein levels (Fig. 3B–M). NLRP3, ASC (mRNA: *Pycard*), CASP1, GSDMD, and IL-1β (mRNA: *IL-1b*) are well-known markers for pyroptosis. Figure 3A shows early increases in pyroptosis-related transcripts in *Rnaset2*^*−/−*^ brains compared to *Rnaset2*^*+/+*^, which declined by 17–28 weeks. *Nlrp3* mRNA was significantly increased at 3 weeks (FC 3.3 ± 0.8; p < 0.05) and decreased at 6 weeks (FC < 2). *Pycard* mRNA was significantly elevated at 3 weeks (FC 3.3 ± 0.2; p < 0.0001) and 6 weeks (FC 2.6 ± 0.2; p < 0.0001), but was not significantly increased at 17 weeks (FC 1.6 ± 0.2). Likewise, the mRNA levels of *Casp1* were significantly increased at 3 weeks (FC 3.9 ± 1.0; p < 0.001) and decreased thereafter (6 weeks: FC 2.4 ± 1.0; 17 weeks: FC 1.8 ± 0.1). *Gsdmd* mRNA levels were significantly increased at 3 weeks (FC 3.5 ± 0.5; p < 0.001), decreased at 6 weeks (FC 2.4 ± 0.5), and remained lower thereafter. *IL-1b* mRNA was elevated at 3 weeks (FC 9.0 ± 2.0), peaked at 6 weeks (FC 16.8 ± 7.0; p < 0.05), and decreased at 17–28 weeks. Taken together, the data demonstrate an initial peak in pyroptosis-related markers at 3 weeks, followed by partial persistence until 6 weeks, and a subsequent decline.

Figure 3B shows increased ASC protein levels *(Rnaset2*^*−/−*^*)* at the 3-week time point. Moreover, ASC band intensities at the other time points differed significantly between *Rnaset2*^*−/−*^ and *Rnaset2*^*+/+*^ mouse brain samples, indicating strong inflammasome activation (Fig. 3E p < 0.05, H p < 0.001, and K p < 0.01). In line with the elevated ASC protein amount described here, we also found a noticeable rise in CASP1 band intensity at 3 weeks, as well as a small increase in GSDMD protein amounts (Fig. 3C, D). Then, CASP1 protein levels in brain samples from *Rnaset2*^*−/−*^ mice showed an apparent but non-significant rise at 6 weeks, while GSDMD protein levels showed a significant increase (Fig. 3F, G, p < 0.05). By 17 weeks, both CASP1 and GSDMD protein levels were significantly elevated (Fig. 3I p < 0.01, J p < 0.001). At 28 weeks, these differences were no longer present, suggesting changes in the regulation of pyroptosis (Fig. 3L and M). Finally, qPCR and protein analyses indicated early gene induction (3–6 weeks) with ongoing inflammasome activation, thereby supporting the involvement of pyroptosis in the neuroinflammatory phenotype of *Rnaset2*^*−/−*^ mice.

### Pyroptosis occurs in IBA-1-positive microglia

**Fig. 4:**
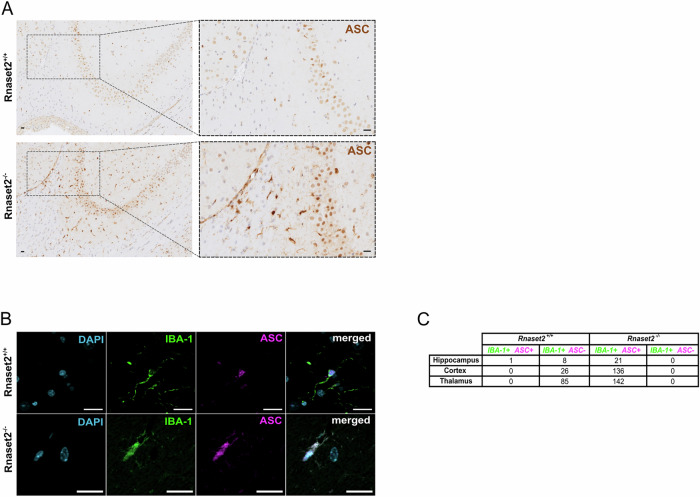
Upregulation of pyroptosis-related proteins occurs in IBA-1 positive microglia. **A** Immunohistochemistry for ASC in a representative brain section from 20-week-old *Rnaset2*^*−/−*^ and *Rnaset2*^*+/+*^ mice revealed an upregulation of ASC in microglia cells of *Rnaset2*^*−/−*^ mice. Scale bar: 20 µm, objective: 10×. **B** Immunofluorescence (IF) co-staining of ASC and IBA-1 confirms intense ASC staining in microglial cells. ASC is more expressed in the cytosol of microglial cells in the *Rnaset2*^*−/−*^ mouse brain compared to *Rnaset2*^*+/+*^ specimens, suggesting the formation of ASC specks. Scale bar: 20 µm, objective: 40×, ROI. (C) IBA + ASC+ and IBA + ASC- cell counting in designated brain regions (hippocampus, cortex, and thalamus) of *Rnaset2*^*−/−*^ and *Rnaset2*^*+/+*^ mice revealed increased IBA + ASC+ cell counts across all brain regions of *Rnaset2*^*−/−*^ mice.

To further investigate pyroptosis in our animal model, we conducted immunohistochemical stainings (IHC) of ASC in brain slices from 20-week-old *Rnaset2*^*−/−*^ and *Rnaset2*^*+/+*^ mice. As illustrated in Fig. 4A, a distinct DAB signal was detected, selectively staining cells of similar sizes and shapes, presumably microglia cells. Subsequently, we performed immunofluorescence (IF) co-stainings of ASC and the microglia marker ionized calcium-binding adapter molecule 1 (IBA-1) to identify the specific cell type undergoing pyroptosis. The merged images in Fig. 4B show major overlaps between ASC and IBA-1 fluorescence signals, suggesting inflammasome-mediated activation of pyroptosis within microglia cells. Moreover, ASC is more prominently localized in the cytosol of microglia from *Rnaset2*^*−/−*^ mice compared to *Rnaset2*^*+/+*^ specimens, indicating the formation of ASC specks. Next, we quantified IBA-1 + ASC+ and IBA-1 + ASC- cells in specific brain regions (hippocampus, cortex, and thalamus, Fig. 4C). We found a notable increase in IBA-1 + ASC+ cells across all brain regions of *Rnaset2*^*−/−*^ mice, suggesting enhanced expression of pyroptosis-related genes in microglia through ASC aggregation in the absence of *Rnaset2*. Taken together, these findings indicate that pyroptosis, rather than apoptosis, plays a crucial role in the neuroinflammatory and neurodegenerative changes in the brains of *Rnaset2*^*−/−*^ mice.

### Sustained ISG induction and microglia pyroptosis is accompanied by early chemokine release and subsequent T cell infiltration

**Fig. 5:**
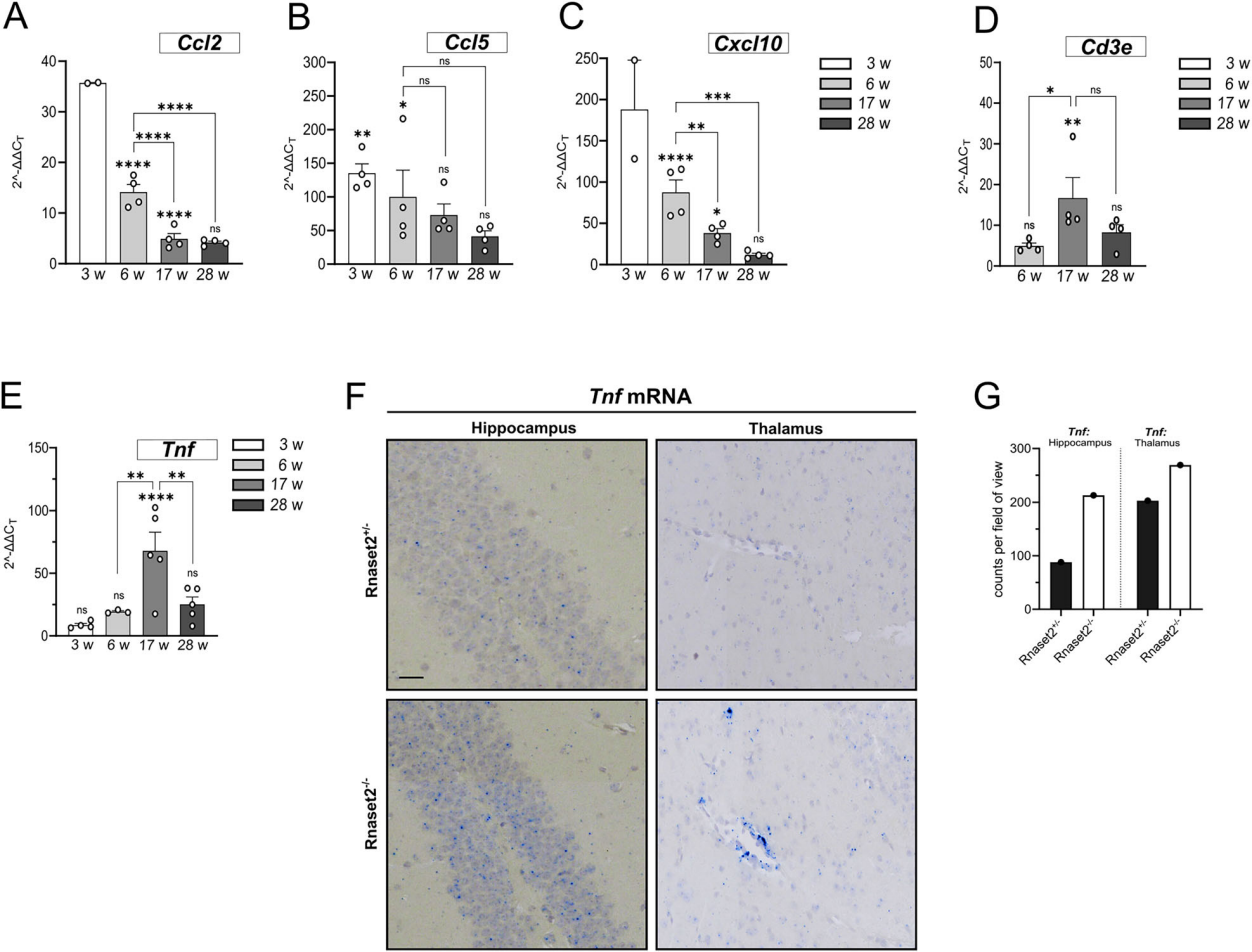
Sustained ISG induction and early pyroptosis precede chemokine release and T cell infiltration. **A**–**D** qPCR analysis of *Ccl2, Ccl5, Cxcl10, and Cd3e* mRNA levels in brain lysates from *Rnaset2*^*−/−*^ mice at 3, 6, 17, and 28 weeks of age, normalized to *Rnaset2*^*+/+*^ controls and a reference gene. The inflammatory chemokines *Ccl2, Ccl5*, and *Cxcl10* were already elevated at 3 and 6 weeks and declined thereafter. The T cell infiltration marker *Cd3e* showed a delayed increase, peaking at 17 weeks (p < 0.01), coinciding with the peak of *Tnf* expression and consistent with early chemokine induction preceding immune cell recruitment (T cell infiltration was absent at 3 weeks; data not shown). Data are presented as scatter dot plots with bars representing mean ± SEM with ^*^ = p < 0.05, ^**^ = p < 0.01, ^***^ = p < 0.001, ^****^ = p < 0.0001, ns not significant; One-way ANOVA followed by Tukey’s multiple comparison test; *n* = *4* per group for all markers and time points, except *n* = *2* at 3 weeks for *Ccl2* and *Cxcl10*. *Ccl2: chemokine (C-C motif) ligand 2, Ccl5: chemokine (C-C motif) ligand 5; Cxcl10: chemokine (C-X-C motif) ligand 10; Cd3e: CD3 antigen epsilon subunit*. **E** Scatter dot plot illustrating alterations in *Tnf* mRNA expression in brain samples from *Rnaset2*^*−/−*^ mice at 3, 6, 17, and 28 weeks of age, normalized to *Rnaset2*^*+/+*^ brain samples and a housekeeping gene, with mean ± SEM. *Tnf* mRNA expression levels fluctuate over time: at 3 weeks, *Tnf* exhibits a mean fold change of 8.6 ± 1.4 (*n* = *4*), increasing to 19.3 ± 0.8 at 6 weeks (*n* = *3*), although not statistically significant. However, a significant peak of *Tnf* mRNA expression occurs at 17 weeks, reaching a mean fold change of 67.89 ± 15.9 (p < 0.0001; *n* = *5*), followed by a significant reduction to 25.3 ± 5.8 at 28 weeks (*n* = *5*), demonstrating a decrease in *Tnf* mRNA content rather than ongoing progression. Data are presented as scatter dot plots with mean ± SEM with ^**^ = p < 0.01, ^****^ = p < 0.0001, ns not significant, One-way ANOVA followed by Tukey’s multiple comparison test; *n* = *4* for 3 weeks, *n* = *3* for 6 weeks, and *n* = *5* for 17 and 28 weeks. *Tnf: tumor necrosis factor alpha*. **F** RNAscope images of *Tnf* mRNA in the hippocampus and thalamus of 26-week-old mice. The quantification of RNAscope dots (blue), which represent individual *Tnf* mRNA molecules, reveals that *Rnaset2*^*−/−*^ mice show a higher *Tnf* expression than *Rnaset2*^*+/−*^ mice. Scale bar: 20 µm, Objective: 20×, ROI, *Tnf: tumor necrosis factor alpha*. **G** Quantification of *Tnf* mRNA. In the hippocampus of *Rnaset2*^*+/−*^, 88 *Tnf* mRNA dots were counted per high-power field, whereas in the hippocampus of *Rnaset2*^*−/−*^, 213 dots were counted. The thalamus contained 203 dots in *Rnaset2*^*+/−*^ and 269 dots in *Rnaset2*^*−/−*^ mouse brain sections per high-power field, indicating that *Tnf* expression is elevated in *Rnaset2*^−^^*/−*^ mice across both brain regions. *Tnf:tumor necrosis factor alpha*.

We demonstrated that *Rnaset2*^*−/−*^ mice develop a severe IFN-I-driven neuroinflammatory disorder, which is characterized by perivascular infiltrates of inflammatory monocytes, activated microglia, and diffuse infiltration of CD8+T cells throughout the entire CNS [11]. Given the sustained ISG upregulation and the observed pyroptosis in microglia, we next investigated whether the associated neuroinflammation follows a similar temporal pattern. The chemokines *Ccl2*, *Ccl5*, and *Cxcl10* showed substantial mRNA upregulation in *Rnaset2*^*−/−*^ brains at 3 weeks and 6 weeks, with declined expressions at 17 and 28 weeks (Fig. 5A–C). Interestingly, the T cell infiltration marker *Cd3e* exhibited a delayed increase (Fig. 5D), reaching its maximum expression at 17 weeks, which corresponds to a peak of *Tnf* expression (Fig. 5E), the known inflammatory marker tumor necrosis factor alpha (TNF-α, mRNA: *Tnf*), which is only detectable at very low levels in healthy individuals and increases upon inflammation [26]. This temporal shift indicates that chemokine production precedes immune cell recruitment, with T cell infiltration becoming most pronounced once inflammation is established. To further examine the spatial distribution and extent of *Tnf* expression, we performed RNAscope with a *Tnf* probe in two brain regions, hippocampus and thalamus (Fig. 5F). The analysis revealed increased counts of *Tnf* mRNA in brain slices from *Rnaset2*^*−/−*^ mice compared to heterozygous controls in both the hippocampus and thalamus (Fig. 5G). Notably, in the thalamus, *Tnf* mRNA counts were especially concentrated around blood vessels, where we also observed IBA-1+ microglia, suggesting a localized inflammatory response in these areas (Fig. 5F).

## Discussion

Type I interferons (IFNs) are central mediators of antiviral defense but can also drive pathological neuroinflammation when chronically activated [27, 28]. Using *Rnaset2*^*−/−*^ mice, a model of intrinsic interferonopathy, we examined the temporal expression of pyroptotic and apoptotic markers and their relationship to ISG activation, chemokine release, and T cell infiltration during interferon-mediated neuroinflammation.

Our data indicate that pyroptosis, rather than apoptosis, is a major contributor to the neuroinflammatory and neurodegenerative phenotype in *Rnaset2*^*−/−*^ mice. Key pyroptotic mediators—ASC, CASP1, and GSDMD—were robustly upregulated at both mRNA and protein levels, peaking between 3 and 6 weeks of age. This developmental window corresponds to the juvenile-to-young adult phase, when microglia mature and acquire homeostatic surveillance functions [29]. Interferon-driven inflammasome activation during this critical period may disrupt microglial differentiation, establishing a persistent state of inflammatory sensitization. Early pyroptotic activation coincided with increased ISG expression and elevated mRNA levels of *Ccl2*, *Ccl5*, and *Cxcl10*, suggesting that pyroptotic signaling amplifies IFN-dependent chemokine release. Microglial numbers were not diminished, supporting a sublytic, activated phenotype characterized by membrane perturbation and mediator release without cell lysis.

The temporal restriction of pyroptotic activation during early development is consistent with heightened innate immune sensitivity of juvenile microglia. Postnatal microglia express elevated inflammasome components and pro-inflammatory mediators, retain open chromatin at IFN- and inflammasome-associated loci, and display a glycolytic metabolic profile favoring inflammatory responses [30–33]. With maturation, microglia acquire homeostatic and anti-inflammatory programs, including TGF-β and neuronal-derived cues, which likely suppress inflammasome responsiveness and decouple chronic interferon signaling from pyroptotic execution [34]. Single-nucleus RNA sequencing confirmed microglia as the principal IFN-responsive cell type in *Rnaset2*^*−/−*^ mouse brains. Although astrocytes, endothelial cells, and neurons also exhibited increased ISG expression, pyroptotic activation was restricted to microglia [11]. This specificity likely reflects both constitutive expression of inflammasome components—ASC, CASP1, and GSDMD—in basal microglia and their specialized sensome, which renders them particularly susceptible to interferon-induced inflammasome activation. Moreover, developmental exposure to IFN-I signaling in the neonatal brain may prime microglia for exaggerated inflammasome responses later in life. Consistent with the importance of microglia dysfunction, the replacement of Rnaset2-deficient microglia with RNASET2-competent macrophages can rescue the neuroinflammatory phenotype in a zebrafish model of RNaseT2-deficiency [35].

### Microglia pyroptosis plays a crucial role in the pathogenesis of neurological diseases

Microglia are crucial in the progression of neurodegenerative diseases, such as Alzheimer’s disease (AD), which is the most prevalent neurodegenerative disorder in the CNS. AD is associated with the accumulation of fibrillar amyloid-β (Aβ) and the formation of neurofibrillary tau tangles. It has been shown that ASC-Aβ complexes can trigger CASP1 activation, IL-1β maturation, and GSDMD cleavage and induce NLRP3 inflammasome formation and pyroptosis in microglia [36]. Furthermore, increased release of ASC exacerbates pyroptosis-mediated neuroinflammatory damage [37]. In addition, tau protein also activates inflammasomes and induces microglia pyroptosis through the NLRP3-ASC axis [38].

The pathological marker of Parkinson’s disease (PD) is α-synuclein, that accumulates in neurons, eventually leading to the formation of Lewy bodies [39]. α-Synuclein, like Aβ, triggers the activation of NLRP3 inflammasomes, leading to the release of ASC from microglia and the occurrence of extracellular ASC patches [40].

CASP1 is activated in the brains of Huntington’s disease (HD) patients and HD mouse models. Moreover, inhibition of CASP1 in HD mouse models was shown to slow down disease progression [41, 42]. Huntington’s protein (HTT) is hydrolyzed and cleaved by CASP1, which produces an N-terminal mutated fragment (mHTT), leading to neuronal dysfunction and death [43, 44]. This finding strongly suggests that pyroptosis might be linked to the pathological generation of HTT. Furthermore, significant NLRP3 activation was observed in microglia of an HD mouse model, indicating a link between neuroinflammation and pyroptosis in microglia [45]. However, NLRP3 upregulation was found in other cell types in the striatum of HD mice but not in microglia by another study [42]. Thus, more in-depth studies are needed to describe pyroptosis in HD.

Type I IFNs act as upstream amplifiers of pyroptotic signaling. In AD models, genetic or pharmacologic blockade of IFNAR1, cGAS, or STING reduces microglial activation, synapse loss, and cognitive deficits [46, 47]. Similar mechanisms are implicated in PD and HD, where mitochondrial stress and DNA release prime microglia for inflammasome activation [48–52]. Finally, in addition to AD, PD, and HD, we demonstrate that microglia pyroptosis occurs in RNaseT2-deficiency, thereby supporting a model in which IFN-I signaling, whether primary or secondary, drives a conserved cascade of inflammasome activation and pyroptotic microglial stress across CNS disorders.

### Targeting pyroptosis as a prospective therapeutic approach for neurodegenerative disorders and neuroinflammation

Pyroptosis offers a promising therapeutic target for the treatment of neuroinflammatory and neurodegenerative diseases. It is reasonable to assume that microglial pyroptosis is not exclusive to RNaseT2-deficient CLE. Given the nearly identical clinical and MRI-morphological changes observed in RNaseT2-deficient CLE and congenital CMV infection, which arise at the same stage of embryonic and fetal neuronal development, microglial pyroptosis is likely to contribute to the resulting brain damage. Furthermore, the presence of a shared key driver—persistent interferon signaling in the brain—along with the clinical overlap with Aicardi–Goutières syndrome, suggests that microglial pyroptosis may play a role in a variety of these disorders.

Moreover, the progression of AD has been closely associated with the systemic activation of inflammasomes and pyroptotic pathways. Therefore, targeting these processes may alleviate cognitive decline and reduce the related inflammatory responses [53]. Recent research has identified several options for addressing pyroptosis. Curcumin usage has been shown to inhibit microglial pyroptosis by impairing the activation of the NLRP3 inflammasome and the NF-κB signaling pathway. This results in a substantial decrease in cognitive impairment and neuroinflammation in animal models [54]. Inhibiting GSDMD, a key effector of pyroptosis, is an additional promising approach. In models of autoimmune encephalomyelitis and other neurodegenerative diseases, researchers have successfully reduced neuroinflammation by blocking GSDMD [53, 55]. Furthermore, neuroprotective effects have been demonstrated by small molecule inhibitors such as MCC950, which precisely target the NLRP3 inflammasome by preventing pyroptotic cell death and subsequent neuronal damage [56–59]. Altogether, these findings indicate that targeting pyroptosis could provide a dual advantage: a reduction in the acute inflammatory response and long-term neuroprotection.

Our study identifies microglial pyroptosis as a central disease mechanism in intrinsic interferon-mediated neuroinflammation and neurodegeneration. Elucidating the molecular events that trigger and sustain this hyperactive microglia state will be essential for developing therapeutic strategies to modulate neuroinflammation across a spectrum of interferon-driven and neurodegenerative disorders.

## Material and methods

### Study design

Owing to limited animal availability and animal-welfare considerations, no formal a priori power analysis was performed. Group sizes were determined in advance using pilot data showing significant effects (≥2-fold mRNA changes and ≥30–50% differences in protein abundance). Accordingly, most key comparisons used *n* = *4* per genotype per time point, which is expected to detect large effects with adequate power, whereas the 3-week time point (*n* = *2*) is reported as descriptive and was not used for inferential testing. Conclusions are supported across orthogonal readouts (qPCR, Western blot, and imaging). Investigators were aware of group allocation (genotype and predefined time points) during sample processing and during outcome assessment. To limit bias, samples from all groups were processed in the same run where feasible, plate/gel positions were balanced between groups, and analysis parameters were pre-specified (e.g., ROUT Q = 1%; identical exposure/quantification settings).

### Breeding and maintenance

*Rnaset2*^−/−^ mice (C57BL/6 N *Rnaset2*a^tm1(KOMP)Wtsi^; *Rnaset2*b^em1Gaer^) and control animals were bred and housed at the Max Planck Institute of Experimental Medicine and the University Medical Center Göttingen, both in Göttingen, Germany. Conditions included 21 °C temperature, 60–65% humidity, a 12 h light/dark cycle, and 2–5 mice per cage. All experiments were performed in accordance with the German animal protection law and with the permission of the Lower Saxony Federal State Office for Consumer Protection and Food Safety (LAVES) under the protocol No. 17-2697.

### RNA extraction, reverse transcription, and quantitative PCR

Two to five mixed-sex *Rnaset2*^*+/+*^ and *Rnaset2*^*−/−*^ mice aged between 3 weeks and 28 weeks were utilized for the extraction of brain RNA with peqGOLD TriFast ^TM^ (VWR, Radnor, PA, USA) according to the manufacturer’s instructions. All mice were perfused with phosphate-buffered saline (PBS, pH 7.5). RNA quality was assessed by measuring the optical density (OD), with subsequent reverse transcription using SuperScript III First-Strand Synthesis System (Thermo Fisher Scientific, Waltham, MA, USA), following the guidelines provided by the manufacturer.

Quantitative PCR (qPCR) was performed using predesigned PrimeTime^TM^ qPCR probe assays (IDT, Coralville, IA, USA), as specified in Table 1. A QuantStudioTM 3 Real-Time PCR System (Thermo Fisher, Waltham, MA, USA) was utilized to conduct qPCR experiments using 20 ng cDNA (40 ng for *Tnf*) synthesized with Oligo(dT) primers (Thermo Fisher, Waltham, MA, USA) in triplicates (SD ≤ 0.20) or in duplicates (SD ≤ 0.15). Housekeeping genes (internal controls) were assessed and selected based on a Ct value difference of ≤1 cycle between *Rnaset2*^*−/−*^ *and Rnaset2*^*+/+*^ samples, following established criteria. Ribosomal protein lateral stalk subunit P0 (*RPLP0)* and TATA-box binding protein (*TBP*) served as internal controls (Table 1). Relative gene expression was quantified using the 2^−ΔΔCT^ method.

**Table 1:** PrimeTime^TM^ qPCR probe assays used for gene expression analysis.

*Targets*	*Sequence name*	*PrimeTime primer 1*	*PrimeTime primer 2*
*Pycard (Asc)*	Mm.PT.56a.42872867	GGT CCA CAA AGT GTC CTG TT	GCT TAG AGA CAT GGG CTT ACA G
*Bad*	Mm.PT.58.41918051	CCC TTC ATC CTC CTC GGT	CAG CCA CCA ACA GTC ATC A
*Bax*	Mm.PT.58.5345963	GTC CCG AAG TAG GAG AGG A	CTA AAG TGC CCG AGC TGA T
*Bcl-2*	Mm.PT.58.7362966	CCA GGA GAA ATC AAA CAG AGG T	GAT GAC TGA GTA CCT GAA CCG
*Bcl2l1 (Bcl-xl)*	Mm.PT.58.30208920	CTC AAC CAG TCC ATT GTC CAA	ATT CAG CAC GAG CAG TCA G
*Bid*	Mm.PT.58.8829163	CCT TGT CGT TCT CCA TGT CTC	GCC GCA CAG TTC ATG AAT G
*Casp1*	Mm.PT.58.8975671.gs	TGC ATC CGT TAA GAA ATC CTC T	GAA AGA CAA GCC CAA GGT GA
*Casp3*	Mm.PT.58.13460531	GGA CTG GAT GAA CCA CGA C	GAC TGA TGA GGA GAT GGC TTG
*Casp8*	Mm.PT.58.41467226	CTC AAT TCC AAC TCG CTC ACT	GGT CAA CTT CCT AGA CTG CAA
*Ccl2*	Mm.PT.58.42151692	AAC TAC AGC TTC TTT GGG ACA	CAT CCA CGT GTT GGC TCA
*Ccl5*	Mm.PT.58.43548565	CCT CTA TCC TAG CTC ATC TCC A	GCT CCA ATC TTG CAG TCG T
*Cd3e*	Mm.PT.58.41881276	CCT TCC TAT TCT TGC TCC AGT	ACG TAC TTG TAC CTG AAA GCT C
*Cxcl10*	Mm.PT.58.43575827	TGA TTT CAA GCT TCC CTA TGG C	ATT TTC TGC CTC ATC CTG CT
*Ddx58*	Mm.PT.58.43881955	TTC CTT GAT CAT GTT CGC CTT	TCT CTA TGA GTA CGT GGG CAA
*Gsdmd*	Mm.PT.58.30932133	CAT CGA CGA CAT CAG AGA CTT	CCG TTA TTC ATG TGT CAA CCT G
*Ifi27l2a*	Mm.PT.56a.41414909.gs	TGA GTT GAG CTG ATA GAA GTG TC	AGG AAG CCT GGT AGC CA
*Ifi44*	Mm.PT.58.43920337	GAT AAG GCA AAA CCA AAG ACT CC	ACG TGG ATA GCC TGG ATC T
*Ifit1*	Mm.PT.58.32674307	TGA AGC AGA TTC TCC ATG ACC	GCA AGA GAG CAG AGA GTC AAG
*Il-1b*	Mm.PT.58.41616450	CTC TTG TTG ATG TGC TGC TG	GAC CTG TTC TTT GAA GTT GAC G
*Irf9*	Mm.PT.58.31054864	ACC ATA GAT GAA GGT GAG CAG	CAA CTG CAA CTC TGA GCT AGA
*Isg15*	Mm.PT.58.41476392.g	CCC CCA TCA TCT TTT ATA ACC AAC	CAC AGT GAT CAA GCA TTT GCG
*Nlrp3*	Mm.PT.58.13974318	CGG TTG GTG CTT AGA CTT GA	CAC TCA TGT TGC CTG TTC TTC
*Rplp0*	Mm.PT.58.43894205	CGC TTG TAC CCA TTG ATG ATG	TTA TAA CCC TGA AGT GCT CGA C
*Rsad2*	Mm.PT.58.11280480	ACG CCA ACA TCC AGA ATA GAC	CCA GAA GAT GAA AGA CTC CTA CC
*Siglec1*	Mm.PT.58.16235548	CAT CCT GAT ACC AGC TGT ATG AG	GTT CAC TCT TCT TCC AGG TCA
*Tbp*	Mm.PT.39a.22214839	CCA GAA CTG AAA ATC AAC GCA G	TGT ATC TAC CGT GAA TCT TGG C
*Tnf*	Mm.PT.58.29509614	ACT TGG TGG TTT GCT ACG A	GAT GAG AAG TTC CCA AAT GGC

PrimeTime^TM^ qPCR probe assays with sequence names and primer sequences.

### Protein extraction and western blotting

Brain hemispheres of 3, 6, 17, and 28 week old *Rnaset2*^*−/−*^ and *Rnaset2*^*+/+*^ mice were obtained as described in Spijker et al. [60]. Lysis was conducted using cell lysis buffer (Cell Signaling, Danvers, MA, USA) supplemented with cOmplete^TM^ and PhosSTOP^TM^ (Merck, Darmstadt, Germany) (solution 1). In brief, homogenization of one brain hemisphere was performed using 2 ml of solution 1, in a Greiner round-bottom tube (Merck, Darmstadt, Germany) with an IKA ULTRA-TURRAX disperser (IKA, Staufen, Germany). Hemispheres were homogenized for 3 × 10 s on ice, followed by a sonication step for 3 × 10 s. Afterwards, samples were rotated at 4 °C for 2 h to enhance protein extraction. Finally, samples were centrifuged at 21255 x g at 4 °C for 20 min, and supernatants were collected and stored at −80 °C until further analysis. The protein concentration was determined using the Interchim BC Assay Kit (Interchim, Montloçon, France), with bovine serum albumin serving as the standard. Protein lysates were denatured by boiling in Tris-Glycine SDS sample buffer, and 20–30 µg were loaded onto 4–20% Tris-Glycine Mini Gels (Thermo Fisher Scientific, Waltham, MA, USA). After performing SDS-PAGE, proteins were transferred onto 0.45 µm nitrocellulose membranes obtained from Merck (Darmstadt, Germany). Next, using 5% non-fat milk in TBS supplemented with 0.1% Tween20 (TBST), membranes were blocked for 1 h at room temperature (RT). Subsequently, primary antibodies were applied in 5% non-fat milk in TBST at 4 °C overnight (Table 2). Caspase 3 control cell extracts (#9663, Cell Signaling, Danvers, MA, USA) were used as positive controls (20 µg per lane). Loading controls were either incubated at 4 °C overnight or for one hour at RT, including mouse anti-β-ACTIN, rabbit anti-β-TUBULIN, and mouse anti-GAPDH (Table 2). Next, membranes were washed with TBST, and either goat anti-rabbit or donkey anti-mouse secondary antibodies conjugated to HRP (Jackson ImmunoResearch, West Grove, PA, USA) diluted 1:5000 in 5% non-fat milk in TBST, were applied for one hour at room temperature (Table 2). After another washing step, Lumi-Light Western Blotting Substrate (Merck, Darmstadt, Germany) or Lumi-Light PLUS Western Blotting Substrate (Merck, Darmstadt, Germany) was utilized to detect the chemiluminescent signal. Western blotting was performed once per target protein due to limited material; all biological replicates for each time point were run on the same gel/membrane. Densitometry was performed across all lanes, and data points represent individual animals (brains). The loading control was probed on the same membrane. Original, uncropped images of full-length membranes (including molecular weight markers) are provided in the Supplementary Material.

**Table 2:** Antibodies used in this study.

*Antibody*	*Order number*	*Company*	*Method*	*Dilution*
IRF9	28845	Cell Signaling, Danvers, MA, USA	Western Blot	1:1000
RIG-I	3743	Cell Signaling, Danvers, MA, USA	Western Blot	1:1000
PARP	9532	Cell Signaling, Danvers, MA, USA	Western Blot	1:1000
cleaved Casp3	9661	Cell Signaling, Danvers, MA, USA	Western Blot	1:1000
cleaved Parp	94885	Cell Signaling, Danvers, MA, USA	Western Blot	1:1000
BAX	Ab32503	Abcam, Cambridge, UK	Western Blot	1:1000
ASC/TMS1	67824	Cell Signaling, Danvers, MA, USA	Western Blot	1:1000
Casp1	24232	Cell Signaling, Danvers, MA, USA	Western Blot	1:1000
GSDMD	15101	Cell Signaling, Danvers, MA, USA	Western Blot	1:1000
β-ACTIN	A5441	Merck, Darmstadt, Germany	Western Blot	1:5000
β TUBULIN	2128	Cell Signaling, Danvers, MA, USA	Western Blot	1:2500
GAPDH	8245	Abcam, Cambridge, UK	Western Blot	1:2500
Rabbit-HRP	111-035-003	Jackson ImmunoResearch, West Grove, PA, USA	Western Blot	1:5000
Mouse-HRP	715-035-150	Jackson ImmunoResearch, West Grove, PA, USA	Western Blot	1:5000
ASC	AG-25B-0006-C100	AdipoGen Life Sciences, San Diego, CA, USA	IF/IHC	1:200
Biotin-SP goat anti-rabbit	111-065-144	Jackson ImmunoResearch, West Grove, PA, USA	IHC	1:200
ExtrAvidin-Peroxidase	E2886	Merck, Darmstadt, Germany	IHC	1:1000
IBA-1	MABN92	Merck, Darmstadt, Germany	IF	1:100
goat anti mouse-AF488	SBA-1090-30	Dianova, Hamburg, Germany	IF	1:100
goat anti-rabbit-AF555	A-21428	Thermo Fisher Scientific, Waltham, MA, USA	IF	1:100

Primary and secondary antibodies with order number, supplier, application, and working dilution.

### Immunohistochemistry

For immunohistochemistry, mixed-sex mice aged 20 weeks were anesthetized with ketamine (250 mg/kg i.p., Ursotamin® (Ketamin), Serumwerk) and medetomidine (2 mg/kg i.p., Cepetor, CP-Pharma). The animals were manually transcardially perfused with phosphate-buffered saline (PBS, pH 7.4) immediately after cardiac arrest and subsequently treated with 4% paraformaldehyde in PBS (pH 7.4). After perfusion, brains were post-fixed in 4% paraformaldehyde in PBS for a minimum of 72 h at 4 °C, embedded in paraffin, and cut into 3–5 µm sections. To evaluate apoptosis-associated speck-like protein containing a CARD (ASC), PFA-fixed, paraffin-embedded tissue sections were incubated overnight at room temperature with a polyclonal rabbit anti-ASC antibody (1:200, AdipoGen Life Sciences, AG-25B-0006). This was followed by sequential 1-h incubations with Biotin-SP-conjugated goat anti-rabbit IgG (1:200, Jackson ImmunoResearch, 111-065-144) and peroxidase-conjugated avidin (1:1000, Sigma-Aldrich, E2886). Immunostaining was visualized using 3,3’-diaminobenzidine (DAB; Sigma-Aldrich, D5637) and imaged with an Olympus SLIDEVIEW VS200 slide scanner.

### Immunofluorescence

Immunofluorescence double-labeling of ASC-positive microglia was performed using primary antibodies against IBA-1 (1:100, Merck, mouse clone 20A12.1) and ASC (1:200, AdipoGen Life Sciences, polyclonal rabbit, AG-25B-0006), followed by AF488-conjugated anti-mouse IgG (1:100, Dianova, polyclonal goat anti-mouse IgG) and AF555-conjugated anti-rabbit IgG (1:100, Thermo Fisher, polyclonal goat anti-rabbit IgG) secondary antibodies. Imaging of IBA-1-ASC co-stainings was conducted using a ZEISS Axio Observer Z1 (Apotome 3) fluorescence microscope using a 40× air objective.

### RNAscope

RNAscope in situ hybridization was performed to detect TNF-α mRNA using the RNAscope® assay (Bio-Techne, Mm-TNFα: 311081) on brain sections from 26-week-old mice, following the manufacturer’s instructions (ACD Bio). Images were acquired using an Olympus SLIDEVIEW VS200 slide scanner.

### Statistical analysis

The number of samples or animals is specified in the caption for each experiment. GraphPad Prism software (GraphPad Software, version 10.5.0, Inc., La Jolla, CA, USA) was used for statistical analysis. Outliers were removed using the ROUT method (Q = 1%), pre-specified a priori; no outlier testing was performed for groups with *n* < *3*. For normally distributed data (Shapiro–Wilk test), a two-tailed Student’s *t* test was used for comparison between two groups (Western blot densitometry). One-way ANOVA with Tukey’s multiple-comparisons test was employed for normally distributed (Shapiro-Wilk test) data when comparing more than two groups. For data not normally distributed, the Kruskal-Wallis test with Dunn’s post hoc test was used for comparing more than two groups. Data are presented as the mean ± standard error of the mean (SEM); *n* refers to the number of brains used. Statistical significance was accepted when p < 0.05.

## Supplementary information

Supplemental Figure Legend

Supplemental Figure

Supplemental Material Western Blots Fig. 1

Supplemental Material Western Blots Fig. 2

Supplemental Material Western Blots Fig. 3 3 and 6 weeks

Supplemental Material Western Blots Fig. 3 17 and 28 weeks

## Data Availability

All data supporting the findings of this study are available within the article and its supplementary material files. Additional raw data are available from the corresponding authors upon reasonable request.
