# Supplementary material for: Unlocking microglia pyroptosis in a model of type I interferon-driven neuroinflammation: lessons from Rnaset2−/− mice
Source: Cell Death Dis. 2025 Dec 27;17(1):138. doi: 10.1038/s41419-025-08350-0 (PMC12848040; doi:10.1038/s41419-025-08350-0)
Supplement: Supplementary file 1 — Supplemental Figure Legend [file 41419_2025_8350_MOESM1_ESM.docx]

**Supplementary Figure**

(A–B) qPCR analysis of interferon-stimulated genes (*Ifi27l2a, Ifi44, Ifit1, Isg15, Rsad2, Siglec1*) in brains of *Rnaset2^-/-^* mice normalized to *Rnaset2^+/+^* and a reference gene at three (A), six, 17, and 28 weeks of age (B). The panel demonstrates a consistent and broad ISG upregulation, indicative of a robust interferon signature in *Rnaset2^-/-^* brains (A-B). Data are shown as scatter dot plots with mean ± SEM. One-way ANOVA with Tukey’s multiple comparison test; ^✱^ = p < 0.05, ^✱✱^ = p < 0.01, ^✱✱✱^ = p < 0.001, ^✱✱✱✱^ = p < 0.0001, ns = not significant. *n=*2 per group for three weeks; *n=4* per group for six, 17 and 28 weeks (except for *Siglec1* at 17 weeks, *n=3*). *Ifi27l2a*: *interferon alpha-inducible protein 27-like 2A (human ortholog: IFI27)*; *Ifi44: interferon-induced protein 44*; *Ifit1:* *interferon-induced protein with tetratricopeptide repeats 1*; *Isg15:* *interferon-stimulated gene 15*; *Rsad2: radical S-adenosyl methionine domain containing 2*; *Siglec1*: *sialic acid binding Ig-like lectin 1.*

(C–F) Western blot analysis of apoptotic markers at three weeks. BAX protein expression was comparable between genotypes, with one *Rnaset2^-/-^* animal showing a minor increase (C). Cleaved CASPASE 3 (cl. CASP3) was undetectable in both genotypes. Caspase 3 control cell extracts (#9663, Cell Signaling Technology) were included as a positive control (D). No differences were observed in full-length PARP (E) or cleaved PARP (F). β-ACTIN or GAPDH served as loading controls. WT1-2: *Rnaset2^+/+^*^,^ KO1-2: *Rnaset2^-/-^*. BAX*:* Bcl-2-associated X protein; cl. CASP3: cleaved caspase-3; PARP: poly(ADP-ribose) polymerase 1; cl. PARP: cleaved poly(ADP-ribose) polymerase 1.
