# Supplementary figures and images for: Unlocking microglia pyroptosis in a model of type I interferon-driven neuroinflammation: lessons from Rnaset2−/− mice
Source: Cell Death Dis. 2025 Dec 27;17(1):138. doi: 10.1038/s41419-025-08350-0 (PMC12848040; doi:10.1038/s41419-025-08350-0)

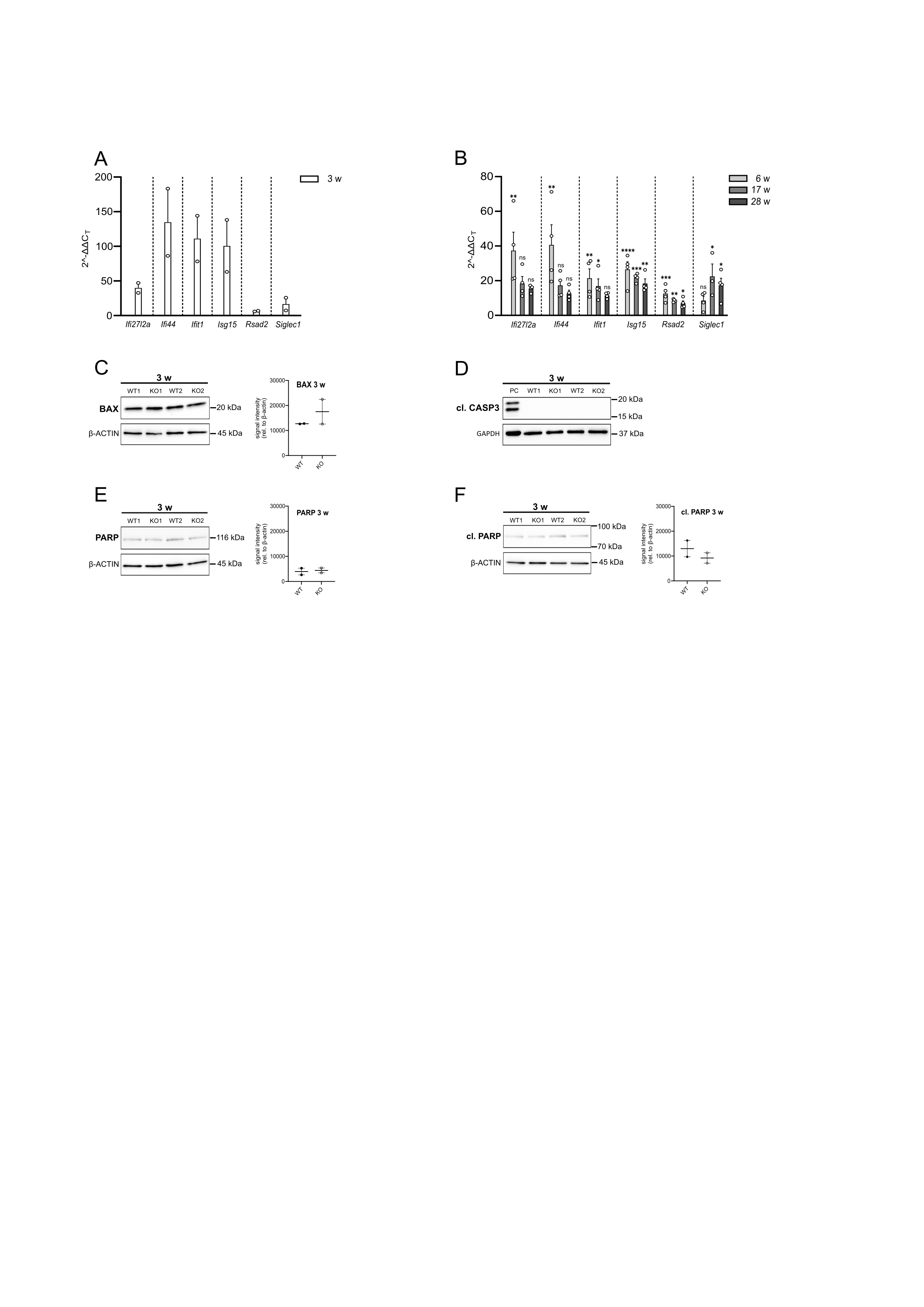

Supplement: Supplementary file 2 — Supplemental Figure [file 41419_2025_8350_MOESM2_ESM.png]

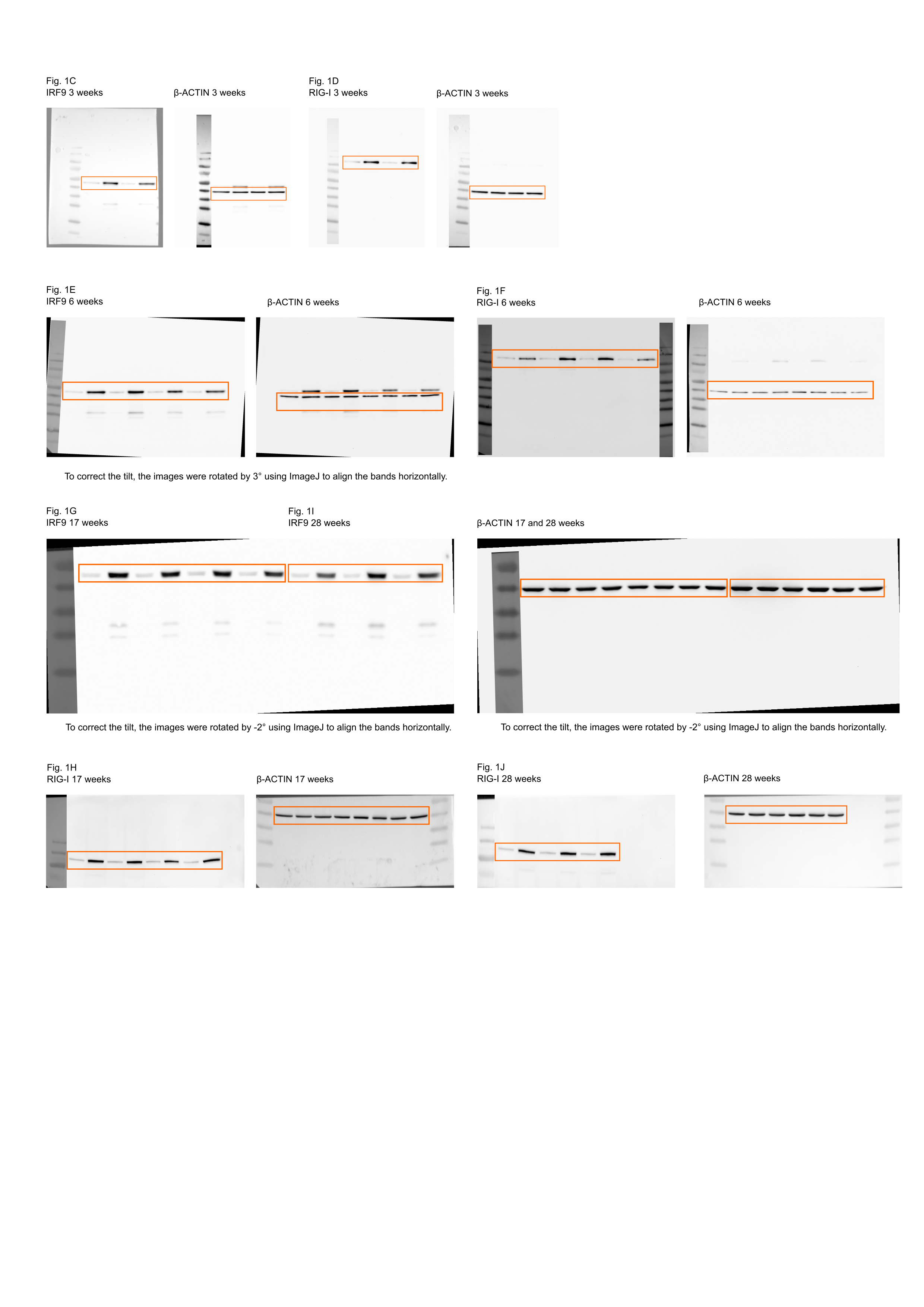

Supplement: Supplementary file 3 — Supplemental Material Western Blots Fig. 1 [file 41419_2025_8350_MOESM3_ESM.png]

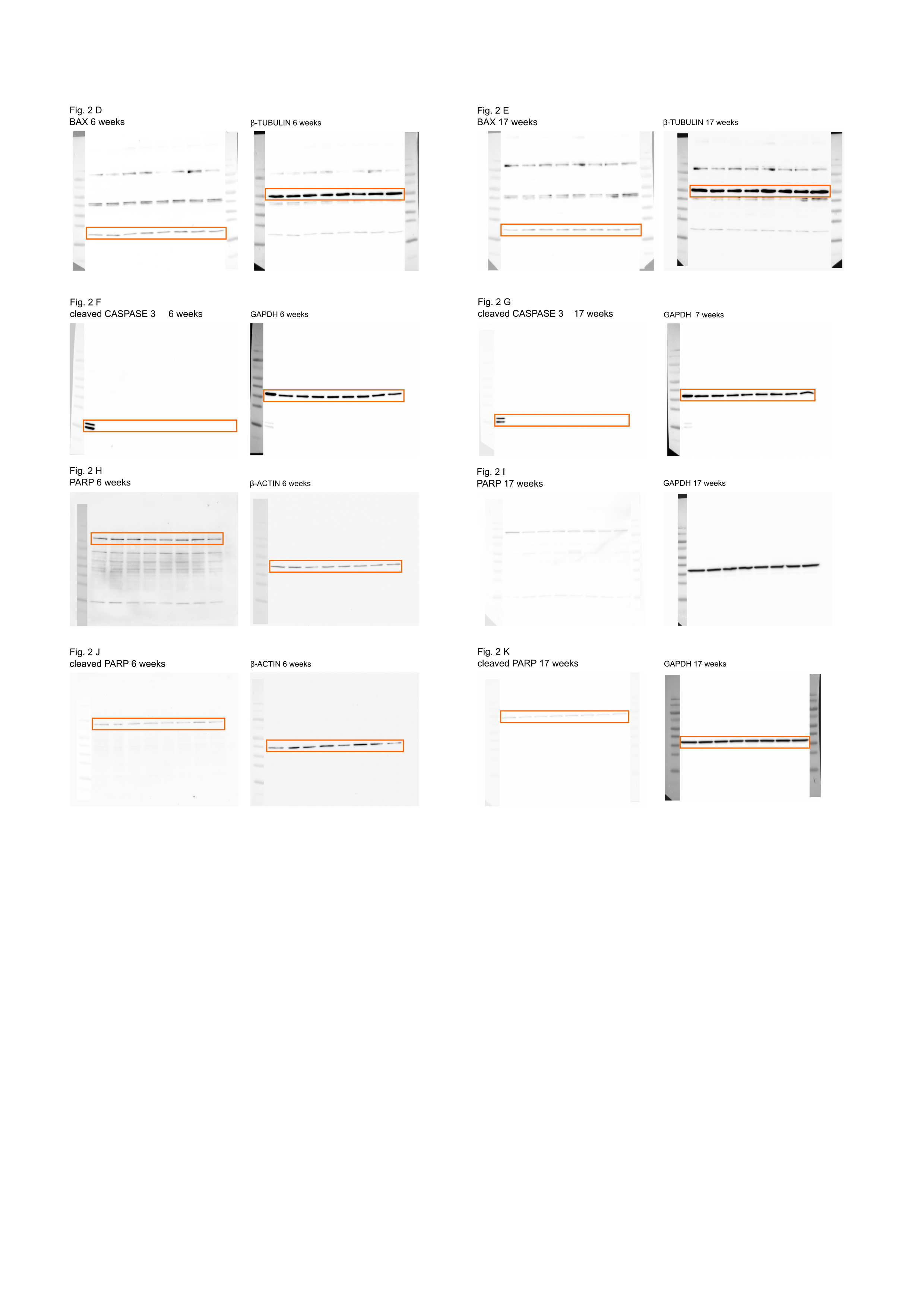

Supplement: Supplementary file 4 — Supplemental Material Western Blots Fig. 2 [file 41419_2025_8350_MOESM4_ESM.png]

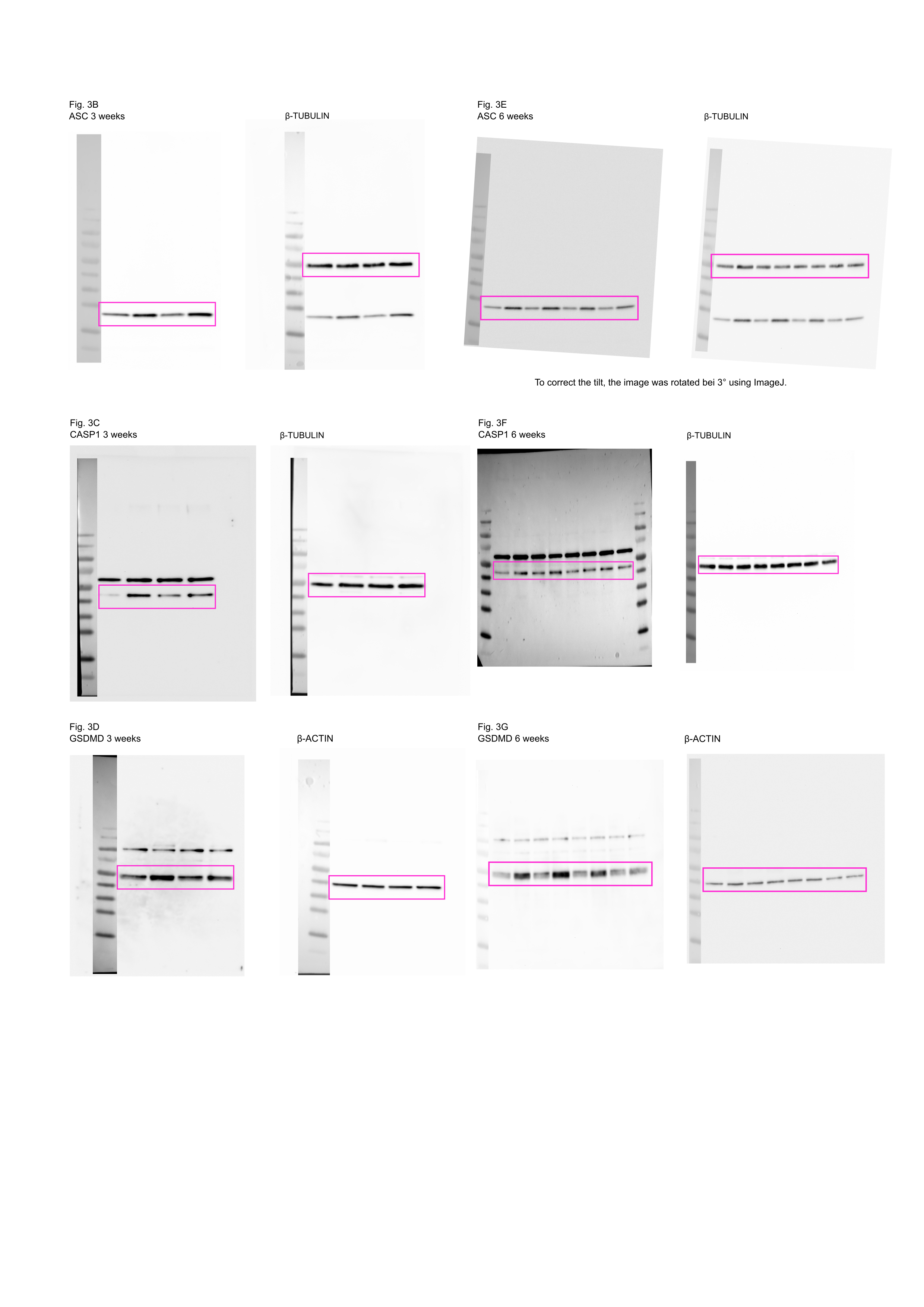

Supplement: Supplementary file 5 — Supplemental Material Western Blots Fig. 3 3 and 6 weeks [file 41419_2025_8350_MOESM5_ESM.png]

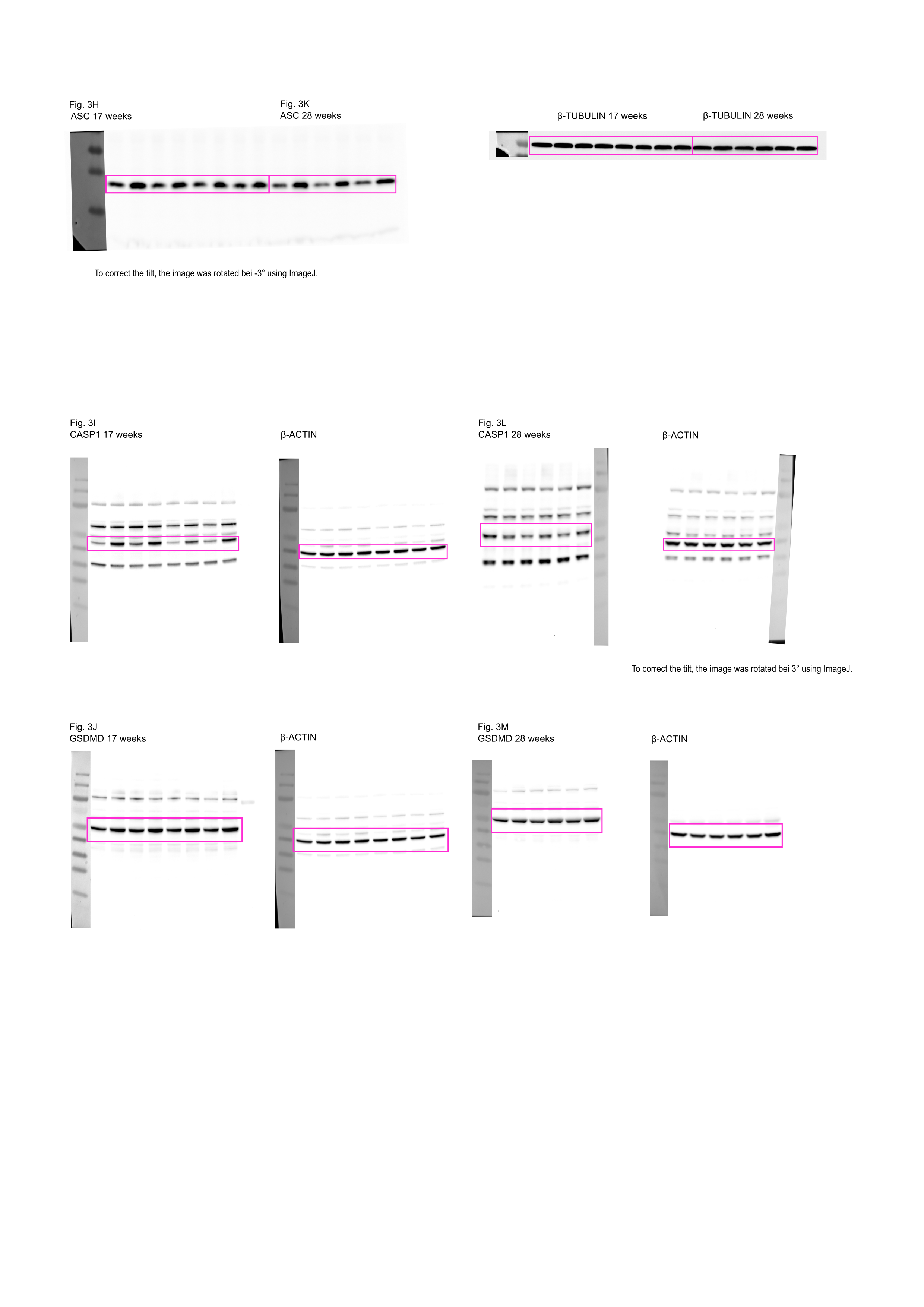

Supplement: Supplementary file 6 — Supplemental Material Western Blots Fig. 3 17 and 28 weeks [file 41419_2025_8350_MOESM6_ESM.png]
